# Deciphering the age-dependent changes of pulmonary fibroblasts in mice by single-cell transcriptomics

**DOI:** 10.3389/fcell.2023.1287133

**Published:** 2023-11-29

**Authors:** Rundong Wu, Xiaowei Zhang, Xinyuan Zhang, Lixiang Sun, Tian Xia, Ling-Juan Zhang

**Affiliations:** State Key Laboratory of Cellular Stress Biology, School of Pharmaceutical Sciences, Xiamen University, Xiamen, China

**Keywords:** pulmonary fibroblasts, myofibroblasts, single-cell transcriptomics, cell interaction, aging, lung fibrosis

## Abstract

**Background and objectives:** The heterogeneity of pulmonary fibroblasts, a critical aspect of both murine and human models under physiological and pathological conditions, is well-documented. Yet, consensus remains elusive on the subtypes, lineage, biological attributes, signal transduction pathways, and plasticity of these fibroblasts. This ambiguity significantly impedes our understanding of the fibrotic processes that transpire in lung tissue during aging. This study aims to elucidate the transcriptional profiles, differentiation pathways, and potential roles of fibroblasts within aging pulmonary tissue.

**Methods:** We employed single-cell transcriptomic sequencing via the 10x Genomics platform. The downstream data were processed and analyzed using R packages, including Seurat. Trajectory and stemness of differentiation analyses were conducted using the Monocle2 and CytoTRACE R packages, respectively. Cell interactions were deciphered using the CellChat R package, and the formation of collagen and muscle fibers was identified through Masson and Van Geison staining techniques.

**Results:** Our analysis captured a total of 22,826 cells, leading to the identification of fibroblasts and various immune cells. We observed a shift in fibroblasts from lipogenic and immune-competent to fibrotic and myofibroblast-like phenotype during the aging process. In the aged stage, fibroblasts exhibited a diminished capacity to express chemokines for immune cells. Experimental validation confirmed an increase of collagen and muscle fiber in the aged compared to young lung tissues. Furthermore, we showed that TGFβ treatment induced a fibrotic, immunodeficient and lipodystrophic transcriptional phenotype in young pulmonary fibroblasts.

**Conclusion:** We present a comprehensive single-cell transcriptomic landscape of lung tissue from aging mice at various stages, revealing the differentiation trajectory of fibroblasts during aging. Our findings underscore the pivotal role of fibroblasts in the regulation of immune cells, and provide insights into why age increases the risk of pulmonary fibrosis.

## Introduction

The lung, a vital organ for gas exchange in humans and most mammals, is prone to various respiratory disorders, including pulmonary fibrosis, pneumonia, and lung cancer, during the aging process (Selman et al., 2016; Selman et al., 2016). Pulmonary fibrosis arises from repetitive and widespread lung injury, in which normal healing process becomes dysregulated and functional tissue is replaced with fibrous scar tissue (Bochaton-Piallat et al., 2016). Pulmonary fibrosis, the most common type of lung diseases, affects nearly five million people worldwide, and studies have shown that approximately one-third of hospitalized COVID-19 patients develop this condition (Vasarmidi et al., 2020; Serra López-Matencio et al., 2021). The relentless scar tissue formed as a result of pulmonary fibrosis can instigate a range of lung diseases, including lung cancer (Liu et al., 2021). However, therapeutic strategies for pulmonary fibrosis remain limited and largely ineffective, and only pirfenidone and nintedanib have been approved to treat patients with progressive pulmonary fibrosis (Hayton and Chaudhuri, 2017).

Aging has emerged as a strongest risk factor for pulmonary fibrosis, as it occurs in middle-aged and primarily elderly adults (Gulati and Thannickal, 2019). The heterogeneous pulmonary fibroblasts (pFBs) play vital role during the progression of fibrosis (Xie et al., 2018). Lung tissues from IPF (idiopathic pulmonary fibrosis) patients display excessive accumulation of ACTA2^+^ myofibroblasts, which can deposit extracellular matrix (ECM) proteins, leading to the destruction of the lung architecture (Gross and Hunninghake, 2001). In the bleomycin-induced murine model of lung fibrosis, lipofibroblasts transdifferentiate into myofibroblasts through activation of the TGFβ signaling pathway (El Agha et al., 2017). How pFBs changes age-dependently has not been characterized. This lack of understanding significantly hinders our comprehension of cellular interactions and molecular signaling pathways among various fibroblast subgroups, as well as the disparities and similarities between age-related and bleomycin-induced pulmonary fibrosis.

Here we aimed to define age-related changes of pFB heterogeneity, differentiation trajectories, and cell-cell communication between pFB and immune cells. Lung tissue cells from mice at different life stages, including 10 days, 2 months, 10 months, and 18 months, were isolated and subjected to single-cell RNA sequencing (Sc-RNAseq) analysis using the 10X Genomics platform. This approach allowed us to assess the diversity of pFBs and refine the existing classification. We further examined characteristic genes, enrichment in signaling pathways, differentiation pathways, cellular interactions, and key transcription factors, thereby constructing a single-cell transcriptomic atlas of aging mouse lung tissue. Results from our study will bring insights into how aging of pulmonary fibroblasts may contribute to age-related increase of the risk of pulmonary fibrosis.

## Materials and methods

### Single-cell RNA library preparation, sequencing, and data processing

For our aging study, we collected whole lung tissues from C57BL/6 male mice at various life stages from neonates, adulthood to old age. Note that we used mice at 18 months of age to characterize old age, because according to Jackson Laboratory, mice ranging from 18 to 24 months of age correlate with human ranging from 56 to 69 years of age (https://www.jax.org/news-and-insights/jax-blog/2017/November/when-are-mice-considered-old). To minimize non-biological batch effects, simultaneous processing, sequencing, and analysis were conducted across all age groups. Lung tissues were minced and digested with collagenase D and DNase1 to isolate single cells, following the protocol as described (Edelman and Redente, 2018). Dead cells were removed using the Dead Cell Removal kit (Miltenyi Biotic, 130-090-101) according to the manufacturer’s instructions. Live cells were then counted with a hemocytometer, resuspended in 2% BSA at a density of 3,000 cells/µL, and processed with the 10x Genomics GemCode Single-cell instrument to generate single-cell Gel Bead-In-EMlusions (GEMs). Barcoded full-length cDNAs were reverse transcribed from polyadenylated mRNA. Silane magnetic beads were used to eliminate residual biochemical reagents and primers post-GEM reaction. Subsequently, cDNA libraries were created, sequenced with Chromium Next GEM Single Cell 3′Reagent Kits v2, and processed on an Illumina Novaseq6000 platform. The raw sequencing data were demultiplexed and aligned to the reference genome mm10-1.2.0 using the Cell Ranger v3.0.2 pipeline (10x Genomics). The resulting raw gene expression matrix was then converted into Seurat objects using the Seurat v2.0 R package. Quality control procedures were implemented to exclude doublets and low-quality cells, including thresholds for gene counts (>200 genes/cell, <5000 genes/cell), unique molecular identifiers (>25,000 UMIs), and mitochondrial gene expression (<8%). As a result, we excluded low-quality cells and outliers, leaving approximately 22,826 viable cells for further downstream analysis. These comprised approximately 5,237 neonatal cells, 4,804 mature adult cells, 6,561 middle age cells, and 6,224 aged cells. For aging samples in mice, we collected lung biopsies from granular tissues. Single cells were isolated and subjected to the same single-cell RNA sequencing (scRNA-seq) procedures, including library construction and data processing, as described above. Low-quality cells and outliers were discarded for downstream analysis. Unsupervised clustering and gene expression visualization were conducted using Seurat 2.0 in R studio. Assignment of cell clusters was performed based on the expression of validated marker genes, including *Dcn* and *Pdgfra* for fibroblasts, *Cd3*, *Cd4*, *Cd8b1*, *Foxp3,* and *Nkg* for T cells, *S100a8* for neutrophils, *Cd68* for macrophages, *Cd19* for B cells. In addition, innate lymphoid cells, pericytes, pneumocytes, and endothelial cells were marked with *Gata3*, *Pdgfrb*, *Sftpb*, and *Ahr*, respectively. To explore the developmental trajectory of lung fibroblasts, pseudotime analysis was conducted on selected pFBs subsets using Monocle 2.10.1 (Qiu et al., 2017). This was followed by the use of scEpath to identify pseudotime-dependent gene expression changes and classify them into distinct differentiation states. CytoTRACE (Cellular Trajectory Reconstruction Analysis) using gene counts and expression, is a computational framework employed for predicting the relative differentiation state of individual cells (Gulati et al., 2020). We utilized R software to estimate transcriptional diversity. To further determine the cell fate of pFBs. To investigate the role of transcriptional regulators during lung aging, we utilized the SCENIC method (Van De Sande et al., 2020). The analysis was conducted in conjunction with the mm9 Rcis Target database, using unique molecular identifiers (UMIs) from transcriptionally defined fibroblasts as inputs to construct regulon networks. These networks highlighted interactions between transcription factors and potential target genes, enabling the scoring of the active values of regulons for each fibroblast cluster. The construction, sequencing, and bioinformatic analysis of single-cell RNA libraries were facilitated by GENE DENOVO Inc (Guangzhou, China).

### Pearson correlation analysis

We conducted Pearson correlation analysis between different scRNA-seq datasets. This was done to assess the correlation or similarity between the pulmonary fibroblast clusters, as identified by scRNA-seq, and lung samples across different ages or various pulmonary fibroblast states. After normalizing and matrixing the scRNA-seq transcriptomic datasets, we computed the Pearson correlation coefficient of the matrix. Subsequently, we performed hierarchical clustering analysis to generate a correlation heatmap using R studio software.

### Cell-chat signaling network analysis

We employed the R package CellChat 1.3.0 (Jin et al., 2021) to evaluate potential intercellular communication between pFB subclusters and other cell types, with a particular emphasis on immune cells, during the aging lung process. scRNA-seq data were processed using the CellChat platform implemented in R software. This procedure encompassed the projection of gene expression data onto a protein-protein interaction (PPI) network, followed by the assignment of probability values to infer biological intercellular communication networks. Additionally, we calculated the centrality indicators of the interactive network to elucidate the role and contribution of each cell population in distinct signaling pathways. The quantity and intensity of identified intercellular communication were visualized through various means such as hierarchical graphs, circular charts, and heatmaps, thus facilitating the display of single or multiple signaling pathways.

### Histology, collagen trichrome staining, and van geison staining

Tissue biopsies were fixed using a 4% PFA solution (Alfa Aesar, Shanghai, China) overnight, followed by dehydration and embedding in paraffin. The paraffin-embedded tissues were then sectioned at a thickness ranging from 5 to 8 mm. For OCT embedding, fresh lung tissues were directly embedded in OCT compound and subsequently sectioned at a thickness of 15–20 mm. Frozen sections were briefly fixed with 4% PFA for 15 min before staining. Histological analysis was conducted using hematoxylin and eosin (HE) staining, utilizing solutions provided by ZSGB-BIO (Beijing, China). Collagen staining was performed using the Masson’s Trichrome Stain Kit (Solarbio, Beijing, China), while Van Geison staining was carried out using the van Geison stain kit (Saint-Bio, Shanghai, China), following the manufacturer’s instructions.

### Primary pulmonary fibroblast isolation and culture

Lung tissues, from 3 weeks male mice, were cut into small pieces and then digested with 2 mg/mL collagenase D, 2 mg/mL dispase I and 1 mg/mL DNase1 for 1.5 h–2 h at 37°C. Cell mixture was filtered through 30 μm filter and treated with red blood cell lysis buffer. Isolated lung fibroblasts were cultured in DMEM supplemented with 10% FBS, glutamax and antibiotics-antimicotics in a humidified incubator at 5% CO_2_ and 37°C under sterile conditions. Primary pulmonary fibroblasts were then trypsinized and replated for *in vitro* assays. To induce *in vitro* fibrosis model, pFBs were treated with recombinant mouse TGFβ2 at a concentration of 3 ng/mL for 2 days (R&D System, 7346-B2-005).

### Quantitative reverse transcription-quantitative PCR (qRT-PCR) analyses

Total cellular RNA was extracted using the RNAExpress Total RNA Kit (NM, M050). Subsequently, 500 ng of RNA was reverse-transcribed to cDNA with the HiScript II Q RT SuperMix kit (Vazyme, R222-01). Quantitative real-time PCR was conducted on the Qtower real-time system (Analytikjena, Swavesey, Cambridge, UK) utilizing the SYBR Green Mix (Bimake, Houston, Texas, United States). Primers used with SYBR Green were designed to span at least one exon, reducing the risk of nonspecific amplification from genomic DNA. The Tbp gene (TATA-Box Binding Protein) served as a housekeeping gene, normalizing data for mouse gene expression. Specific primer sequences are provided in Supplementary Table S2.

### Quantification and statistical analysis

Experiments were conducted a minimum of three times, yielding consistent results, and were analyzed using GraphPad Prism 9 software. For experiments involving two groups, the Student’s unpaired two-tailed *t*-test determined statistical significance. The Shapiro-Wilk test assessed normality. For non-normally distributed datasets, nonparametric tests ascertained statistical significance. A *p*-value less than 0.05 was deemed statistically significant (*p < 0.05, **p < 0.01, ***p < 0.001, ****p < 0.0001).

## Results

### Commencing with the classification of cells within aging mouse lung tissue via scRNA-seq

**FIGURE 1:**
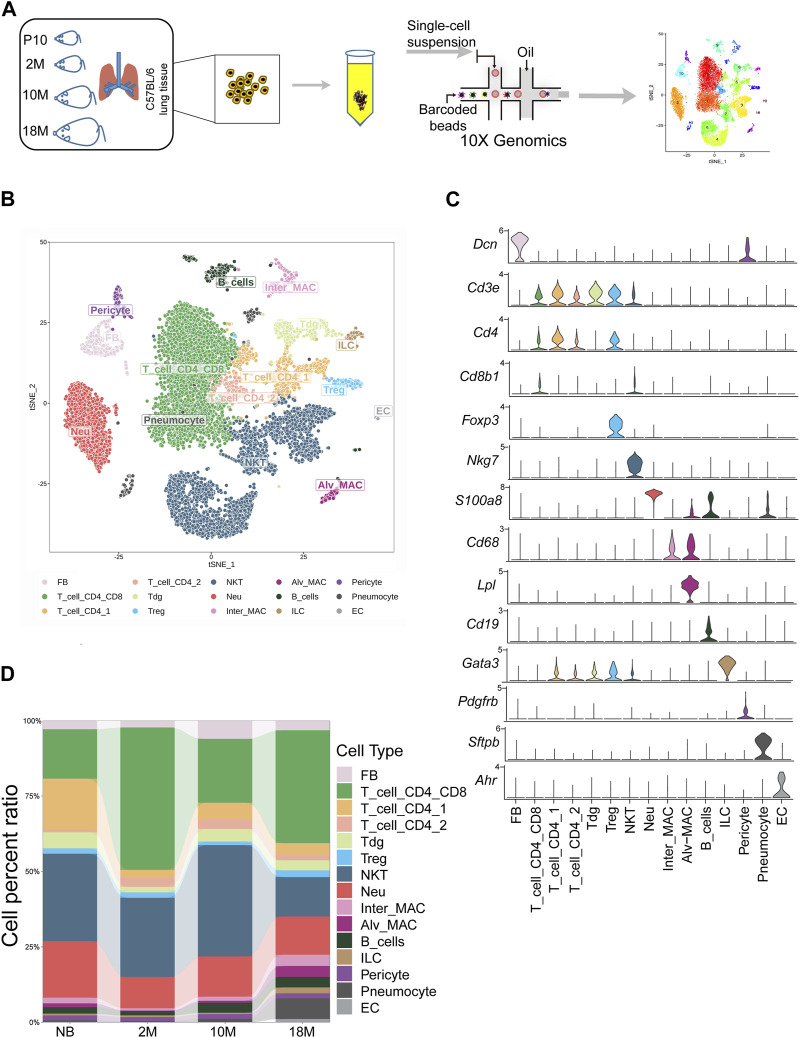
Commencing with the classification of cells within aging mouse lung tissue via scRNA-seq. **(A)**. Schematic diagram of mouse subjected to aging lung tissue. P10, postnatal day 10; 2 M, 2 months years old; 10 M, 10 months years old; 18 M, 18 months years old. **(B)**. tSNE projection of all 22,826 sequenced mouse cells, showing the partitioning of 15 cell types by scRNA-seq. **(C)**. Violin plot showing special marker genes for each cell type in the mouse lung dataset. **(D)**. Bar chart for proportions of the 15 major cell types in lung tissues.

In our study, we utilized scRNA-seq to categorize cells within the lung tissue of C57BL/6 mice at various stages of life (Figure 1A): newborn (NB, post-natal day 10), mature adult (2 months old, 2 M), middle-aged (10 months old, 10 M), and aged (18 months old, 18 M). The lung tissues were processed, and single cells were isolated through enzymatic digestion and tissue fragmentation. These isolated cells were then subjected to single-cell transcriptomic sequencing using the 10X Genomics platform, capturing a total of 22,826 cells. We employed t-distributed stochastic neighbor embedding (t-SNE) for dimension reduction, which facilitated a two-dimensional visualization of cellular expression profiles. The lung tissues were found to comprise 15 cellular sub-clusters, which were classified into 9 cell types, including fibroblasts, T cells (including CD4^+^, CD8^+^, δγ T, Treg (regulatory T cells), and NKT (natural killer T), neutrophils, macrophages, B cells, ILCs (innate lymphoid cells), pericytes, endothelial cells (EC), and alveolar cells (pneumocytes), based on their respective marker genes (Figures 1B, C; Supplementary Figures S1A–C) (Guo et al., 2015). The fibroblast population represented 3.67%, T cells 70.33%, neutrophils 13.9%, macrophages 3.78%, B cells 2.72%, Langerhans cells 0.99%, pericytes 1.58%, alveolar cells 2.63%, and endothelial cells 0.35% of the total cells (Figure 1D). Together, we have performed unsupervised clustering results of mouse lung tissues through life-span, allowing us to investigate the dynamic changes of lung tissue subpopulations throughout aging further.

### Transcriptional characteristics associated with the aging process of lung fibroblasts

**FIGURE 2:**
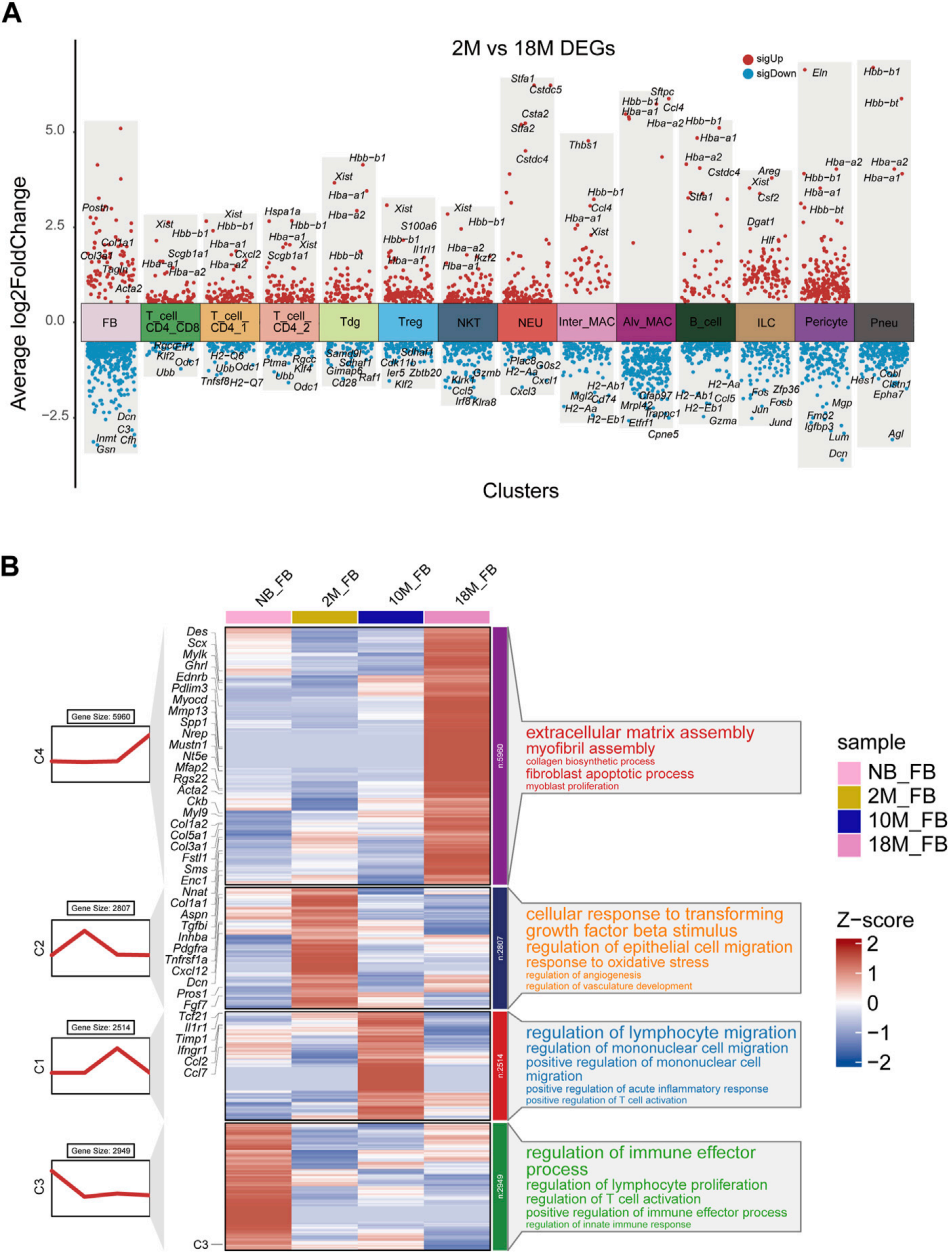
Transcriptional characteristics associated with the aging process of lung fibroblasts. **(A)**. Volcano plot for aging differentially expressed genes were changed in 18 M group compared to the 2 M group for major cell types by Seurat analysis. Red, upregulated (LogFC > 0.5, adjusted *p*-value < 0.01); blue, downregulated group (LogFC < −0.5, adjusted *p*-value < 0.01); **(B)**. Representative aging specific gene modules and pathways enriched in aging differentially expressed genes based on GO-biological process functional enrichment analysis.

We next the characterized how aging changes the transcriptional profiles of pFBs. Differential gene expression analysis comparing key cell clusters from the aged 18 M and young-adult 2 M-old mice showed that aged pFB expressed higher levels of collagen genes (*Col1a1, Col3a1*), and fibrosis-associated marker genes (*Acta2, Postn, Tagln*) (Figure 2A). Previous research has recognized *Pdgfra*, *Acta2,* and *Col1a1* as markers for pulmonary myofibroblasts (Kheirollahi et al., 2019; Peyser et al., 2019; Liu et al., 2021) Periostin (*Postn*) has been observed to be significantly upregulated in the IPF model (Nance et al., 2014). Moreover, protein quantification from IPF revealed substantial changes in *TAGLN* (Tian et al., 2019). Differential gene expression analysis also showed that markers of normal fibroblast function, such as *Dcn* (Souma et al., 2018), were notably lost in the aged pFBs (Figure 2A). We have also presented the top five significantly differentially regulated genes, both upregulated and downregulated, in other key cell types, such as T cells, myeloid cells, and pneumocytes (Figure 2A; Supplementary Table S2). A comprehensive analysis of the fibroblast population at distinct aging stages (Figure 2B) revealed that the 18 M aged pFBs exhibited differential expression of various myofibroblast marker genes, including *Des* (Bär et al., 2004), *Scx*, *Aspn*, *Mustn1* (Xie et al., 2018), *Mylk*, *Ednrb*, *Pdlim3*, *Myocd*, *Nrep*, *Nt5e*, *Acta2*, *Mfap2*, *Ckb,* and *Myl9* (Guo et al., 2015). Concurrently, 18 M pFBs also expressed higher levels of several collagen-related genes, such as *Col1a1*, *Col1a2*, *Col3a1*, and *Col5a1* (Figure 2B). Further analysis revealed an enrichment of differentially expressed genes in 18 M pFBs within signaling pathways related to extracellular matrix assembly, myofibril assembly, collagen biosynthesis, fibroblast apoptosis, and myoblast proliferation (Figure 2B). On the other hand, in 2 M and 10 M fibroblasts expressed higher levels of chemokines such as *Ccl2*, *Ccl7,* and *Cxcl12*, and immune-associated receptors such as *Il1r1*, *Ifngr1*, and *Tnfrsf1a*. Distinct immunomodulatory and angiogenic functions were also observed in the newborn and 10-month-old fibroblasts, and newborn fibroblasts were enriched with pathways related to regulation of lymphocyte proliferation, T cell activation, and innate immune responses, while 2 M and 10 M fibroblasts showed responses to *TGFβ2* stimulation, epithelial cell migration, oxidative stress, vascular development, lymphocyte migration, monocyte migration, and acute inflammatory response regulation, respectively (Figure 2B). In summary, our results indicate that aging of pulmonary tissue is concomitant with a conversion of pFBs from non-fibrotic and immuno-competent to a immuno-deficient and myofibroblast-like, collagen-producing pro-fibrotic phenotype. The fibrotic change of pFBs may instigate fibrosis, thereby contributing to increased risk of pulmonary fibrosis following lung injury in aged individuals.

### Characteristics of fibroblast aging in tissue

**FIGURE 3:**
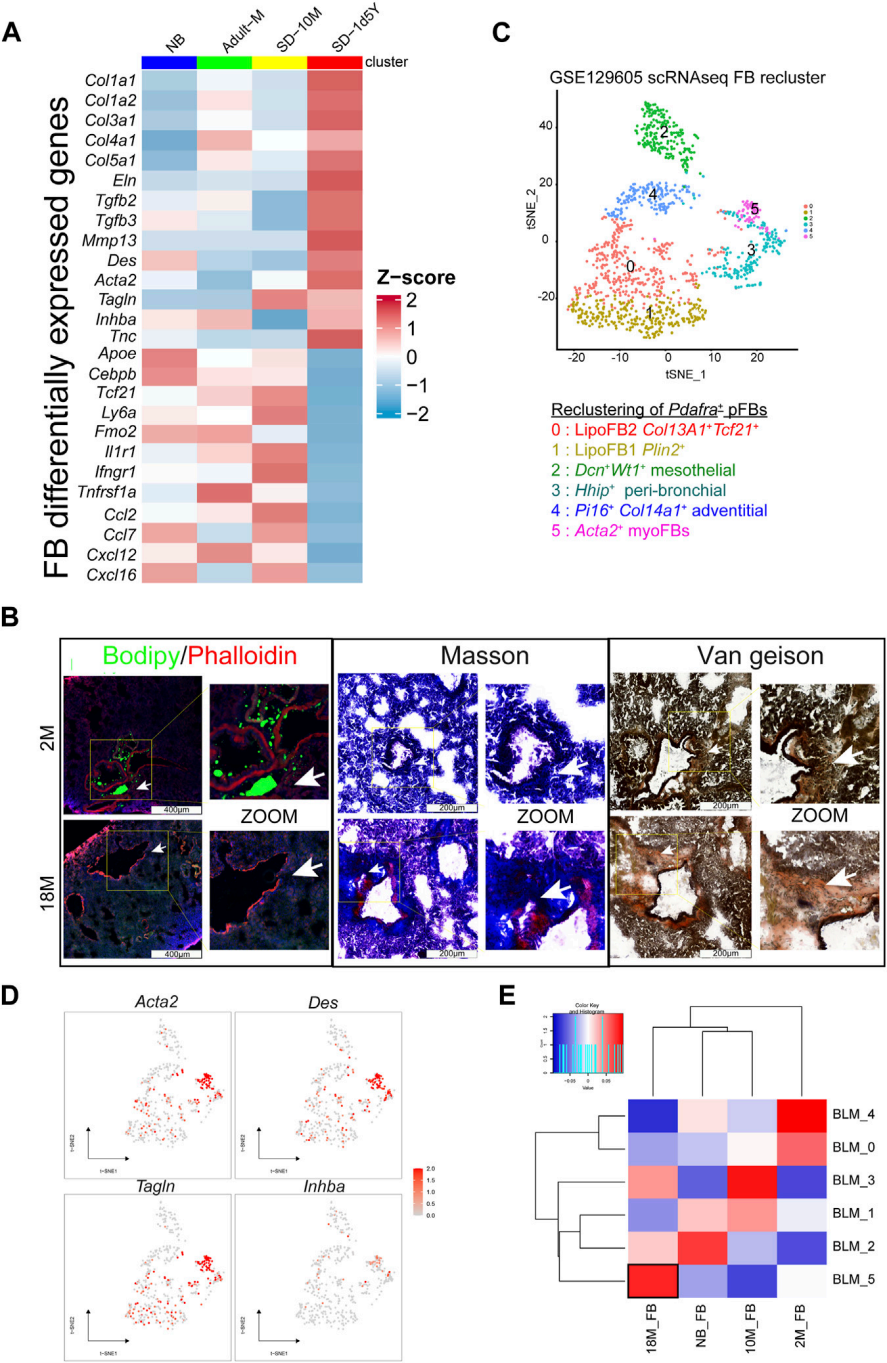
Tissue characteristics of aging fibroblasts. **(A)**. Heatmap showing the variable genes in aging mouse lung pFBs by z-score normalization analysis. Color scale of red to blue indicates z-score. **(B)**. Lipid stain/Masson stain/Van Geison stain showing tissue characteristics changes in 18 M group compared to the 2 M group of lung tissue. Scale bars, 200 μm and 400 μm. **(C)**. tSNE plot for BLM model pFB reclusters by scRNAseq analysis. **(D)**. Feature plot showing on tSNE projection of pFB reclusters. **(E)**. Heatmap showing BLM scRNAseq pFBs reclusters and aging pFBs reclusters relevance by pearson correlation analysis.

Through detailed examination of the transcriptomic changes of fibroblasts in healthy mice across various aging stages, we found that aged pFB displayed a myofibroblast-like profibrotic phenotype, characterized by elevated *Acta2* and collagen gene expression, a phenotype similar to pFBs from bleomycin-treated fibrotic lung tissues. It is well-documented that myofibroblasts, a specialized type of activated fibroblasts, play a crucial role in fibrosis by depositing extracellular matrix (ECM) proteins within scar tissue (Hung, 2020). Single-cell expression data, normalized and visualized via a heatmap, revealed that compared to young pFBs, the 18 M aged pFBs expressed higher levels of *Acta2* and a panel of collagen-associated genes (*Col1a1*, *Col1a2*, *Col3a1*, *Col4a1*, *Col5a1*). This was accompanied by a significant decline in the expression of lipogenesis genes (*Apoe, Cebpb, Tcf21*, and *Ly6a*), cytokine/chemokine receptors (*Il1r1*, *Ifngr1*, *Tnfrsf1a*) and chemokines (*Ccl2*, *Ccl7*, *Cxcl12*, and *Cxcl16*) (Figure 3A). To validate the age-related loss of lipogenesis and increase of collagen expression, we applied lipid and collagen stainings on lung tissue sections from mice at 2 M and 18 M. Bodipy (lipid) staining showed that numerous lipid-droplets can be detected around the peri-bronchial and per-vessel regions of the air way in the young adult but not in the aged lung samples (Figure 3B). In contrast, collagen was heavily deposited around the air way of the aged lung tissue as shown by masson and van geison staining (Figure 3B). These results demonstrated that an adipogenic to fibrotic switch of pulmonary fibroblasts occurred during aging, contributing to loss of lipid and gain of collagen fiber around the trachea following tissue aging.

To determine the similarity between aged pFBs and the myofibroblasts in fibrotic lung tissue, we analyzed a publicly available database (GSE129605), containing single-cell data from the bleomycin (BLM)-induced murine pulmonary fibrosis model (Peyser et al., 2019). We reclustered *Pdgfra* ^+^ pFBs from BLM-treated mice into 6 clusters (BLM_0 ∼ BLM_5) (Figure 3C). According to the differentially expressed marker genes and their distribution, groups 0 and 1 were defined as lipoFB (*Col13a1* ^*+*^ *Tcf21* ^*+*^ *Plin2* ^*+*^), group 2 as mesothelial FB (*Wt1* ^*+*^ *Dcn* ^*+*^), group 3 as peri-bronchial FB (*Hhip* ^*+*^), group 4 as adventitial FB (*Col14a1* ^*+*^ *Pi16* ^*+*^), and group 5 as myoFB (*Acta2* ^*+*^ *Tagln* ^*+*^). (Figure 3C; Supplementary Figure S2). The pFB BLM_5 cluster highly expressed myofibroblast marker genes, including *Acta2*, *Des*, *Tagln,* and *Inhba*, and thus was defined as myofibroblasts (Figure 3D). Correlation analysis revealed that the 18 M aged pFBs highly corelated with the BLM_5 myofibroblasts (Figure 3E), supporting our finding that aging promotes the conversion of pFBs towards a myofibroblast-like phenotype, which may contribute to increased risk for pulmonary fibrosis of the aged individuals.

### The differentiation potential of fibroblasts in lung tissue during aging

**FIGURE 4:**
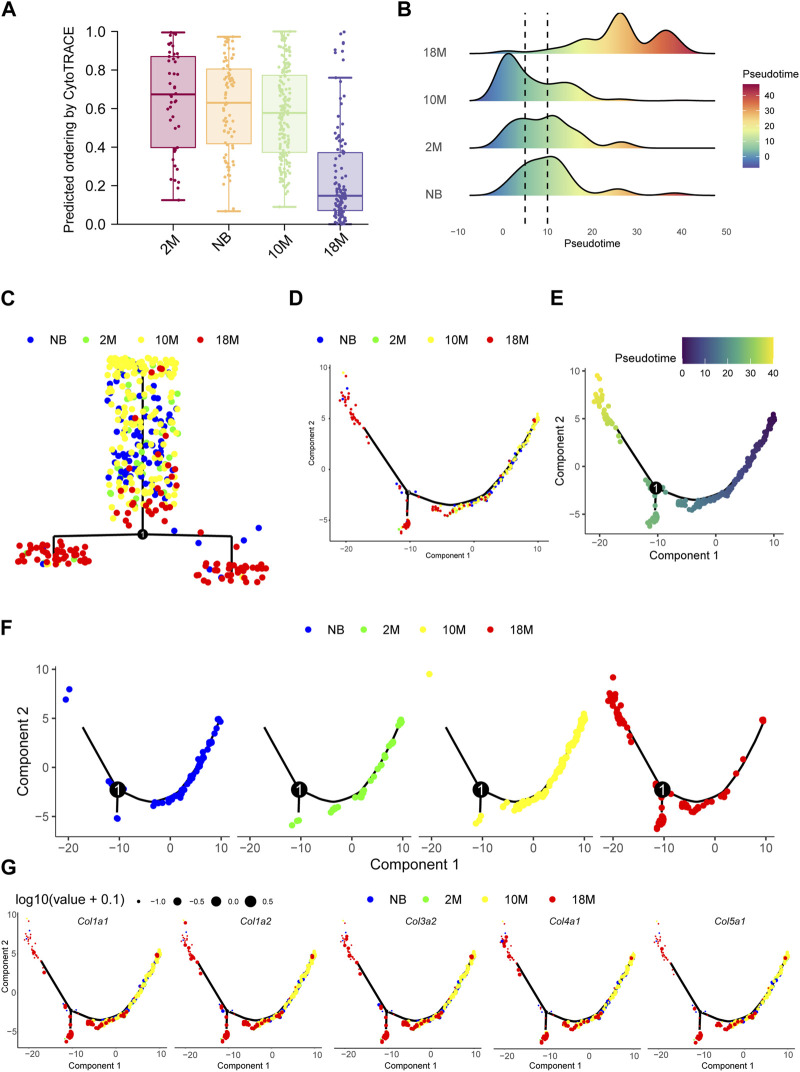
The differentiation potential of fibroblasts in lung tissue during aging. **(A)**. Bar chart for aging pFBs stemness of differentiation by CytoTRACE analysis. **(B)**. Pseudotime density showing the differentiation of fibroblasts from normal fibroblasts to myofibroblasts by Monocle2 analysis. **(C)**. Complex trajectory plot showing the branch of aging pFBs. **(D)**. Pseudotime trajectories showing cell states in all group. **(E)**. Pseudotime trajectories gradients showing differentiation direction of pFBs**. (F)**. Pseudotime trajectories showing cell states splite by each group. **(G)**. *Col1a1*, *Col1a2*, *Col3a1*, and *Col5a1* expression on cell pseudotime trajectory.

CytoTRACE analysis (Gulati et al., 2020) showed that as mice aged, pFBs displayed a progressive decline in differentiation potential and a marked decrease in stemness of differentiation (Figure 4A). To elucidate the relationship between the differentiation of lung fibroblasts and myofibroblasts during the aging process in mice, we subjected our samples to pseudotime analysis and executed gene clustering predicated on pseudotemporal expression patterns. The data compellingly demonstrate that myofibroblasts emerge as the ultimate differentiation trajectory for NB, 2 M, 10 M, and 18 M fibroblasts throughout the aging process (Figures 4B–G), thereby substantiating that a substantial proportion of fibroblasts undergo differentiation into myofibroblasts as aging ensues.

### Predicting the activation state of transcription factors in fibroblasts at different aging states

**FIGURE 5:**
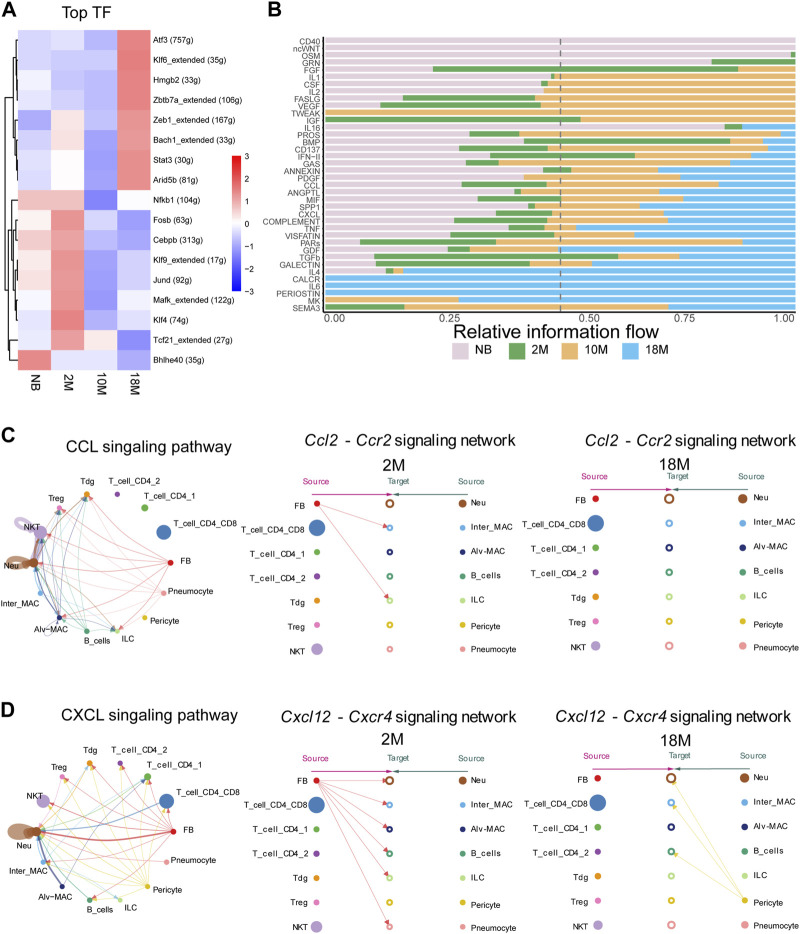
Anticipating the communication dynamics between fibroblasts and immune cells at various stages of aging. **(A)**. Heatmap for significant regulons for each pFBs by SCENIC analyze. **(B)**. Bar chart showing significant signaling pathways were ranked based on their differences in overall information flow within the inferred networks between aging lung tissue by cellchat analysis. **(C)**. Circle and hierarchy plot showing total, 2 M, 18 M cell-cell communication in CCL signaling pathway (left, middle, right). **(D)**. Circle and hierarchy plot showing total, 2 M, 18 M cell-cell communication in CXCL signaling pathway (left, middle, right).

We next performed analysis to predict the activation state of transcription factors (TFs) in pFBs across diverse aging stages, which may bring insight into mechanisms underlying age-related changes of pFB. As shown in Figure 5A, several TFs, including Atf3, Klf6, Zeb1, Bach1, and Stat3, were identified highly active TFs in aged pFBs, It has been shown that ATF3 expression increases in the lung with age (Bueno et al., 2018), and Zeb1 (zinc-finger E-box binding homeobox 1) is significantly upregulated in IPF and is considered as a new therapeutic target for IPF (Qian et al., 2019). In addition, KLF6 has been found to regulate the epithelial-to-mesenchymal transition process in diabetic pulmonary fibrosis (Zou et al., 2017). Furthermore, levels of phosphorylated STAT3 are elevated in lung tissues from patients with IPF and from BLM-treated mice, and STAT3 inhibitor can decrease fibroblast-to-myofibroblast differentiation (Pedroza et al., 2016). On the other hand, activation of Bach1, a transcript factor predominantly implicated in the antioxidant response under pulmonary conditions, could potentially augment age-related oxidative stress in the lung, thereby hastening the progression of aging and fibrosis (Zhang et al., 2018). Our results suggest that these transcription factors may play important regulatory roles driving the development of fibrotic phenotypes of pFBs during aging.

### Characterization of the communication dynamics between fibroblasts and immune cells at various stages of aging

Alterations in intercellular communication emerge as key hallmarks of aging (López-Otín et al., 2013), therefore we next aimed to determine the communication dynamics between fibroblasts and immune cells across diverse aging states. We employed CellChat, a tool engineered to unravel intercellular communication networks through ligand-receptor interactions (Jin et al., 2021). We compared the overall intercellular (Figure 5B) and incoming and outgoing signal intensities (Supplementary Figures S3A–C) within disparate signaling pathways among NB, 2 M, 10 M, and 18 M lung tissues. In the course of aging, we have identified a notable alteration in the intensity of interactions among communities. Subsequently, we performed an in-depth examination of the specific ligand-receptor interaction pairs within these signaling pathways. In the signaling pathways associated with pFBs, a marked alteration has been observed in the interaction intensity of specific ligand-receptor pairs within CCL, CXCL, IGF, Complement, and PROS during the course of aging. Especially, It is essential to emphasize that within the CXCL and CCL signaling pathway, our data identified fibroblasts as the primary sources of Ccl2 and Cxcl12 (Figure 5C; Supplementary Figures S4A, B). Binding of *Ccl2* to *Ccr2* receptor present on macrophage surfaces can potentiate macrophage migratory ability, and foster macrophage recruitment to the inflamed tissue site (Galipeau, 2021; Lei et al., 2021). Here, cell chat analysis revealed that the pFB-MAC interaction through the CCL2-CCR2 signaling network was largely lost in 18 M aged cells. The Cxcl12-Cxcr4 signaling axis plays a vital role in lung tissues, orchestrating a range of biological processes including the recruitment and retention of macrophages at sites of inflammation, which in turn influences the progression of inflammatory diseases. Furthermore, this signaling pathway is involved in the migration of fibroblasts to the lungs, facilitating tissue repair, and has been linked to pathological changes in conditions such as pulmonary fibrosis (Xie and Zhao, 2017; Isles et al., 2019; Jaffar et al., 2020; Tang et al., 2021). Cell-chat analysis showed that in the 2 M-aged cells, *Cxcl12* signal originated from pFBs, whereas in the 18 M-aged cells, Cxcl12 expression was lost in pFBs and in turn was primarily produced by pericytes (Figure 5D). The *Pros1*-*Axl* signaling pathway regulates apoptosis and inflammatory responses during lung tissue aging, contributing to pulmonary stability by restraining excessive immune responses, thereby preventing severe damage, inflammation, and subsequently slowing lung aging (Waterborg et al., 2018). Our analysis revealed that *Pros1* was secreted from pFBs acting on alveolar macrophages, and this interaction was intensified in the 18 M-aged compared to 2 M groups (Supplementary Figure S5A). This aligns with the self-protection mechanism associated with myofibroblast formation and the marked reduction in immune responses during aging. The *Igf1*-*Igf1r* pathway is pivotal in regulating cell proliferation, differentiation, and apoptosis, as well as maintaining the structural and functional stability of lung tissue during repair and regeneration, thus critically contributing to the prevention of lung damage, inflammation, and deceleration of the aging process (Kineman et al., 2018; Cottage et al., 2019; Sun et al., 2021). Our data indicated that Igf1 was originated from fibroblasts and pericytes in the 2-month group, and this signal subsequently declined during aging (Supplementary Figures S5B). These results indicate that the intercellular communication between pulmonary fibroblasts and immune cells become dysregulated during aging.

### Activation of TGFβ pathway induces the transcriptional signature of aging in cultured pulmonary fibroblasts

**FIGURE 6:**
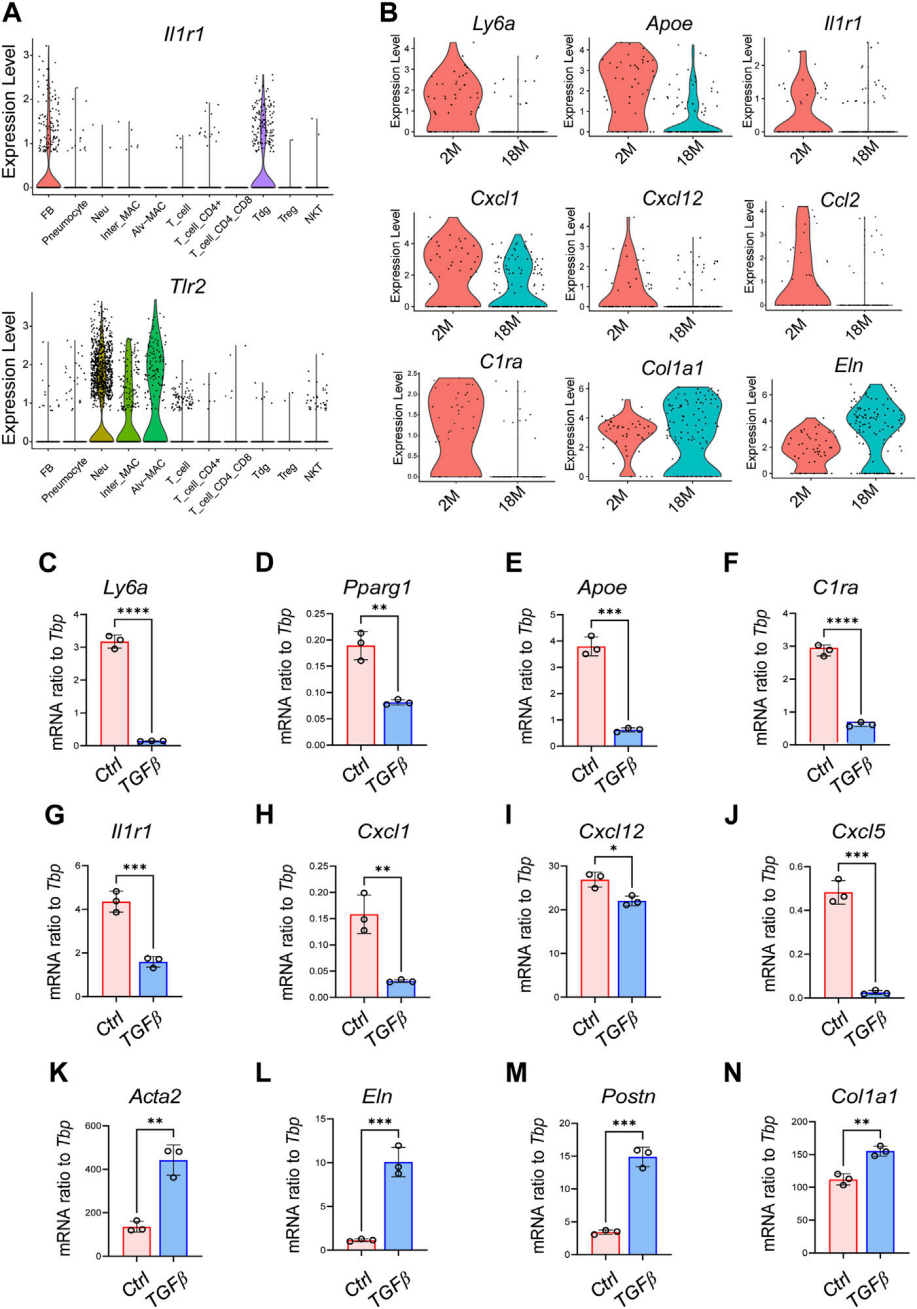
Activation of TGFβ pathway induces the transcriptional signature of aging in cultured pulmonary fibroblasts. **(A)**. Violin plots showing the expression of Il1r1 and Tlr2 in various pulmonary cell clusters as shown. **(B)**. Violin plots showing changes of genes related to lipogenesis (Ly6a, Apoe), immune (*Il1r1, Cxcl1, Cxcl12, Ccl2, and C1ra*), and fibrosis (*Col1a1, Eln*) in pulmonary fibroblasts from 2-month and 18-month mice. **(C–N)**. Primary pulmonary FBs were treated with recombinant mouse TGFβ at a concentration of 3 ng/mL for 2 days and then subjected to qRT-PCR of indicated genes (*n* = 3/group). All error bars indicate mean ± SEM; *p < 0.05, **p < 0.01, ***p < 0.001.

Pulmonary innate immunity plays a critical role in protecting the lung from airborne pathogens, and age-related loss of pulmonary innate immune function contributes to increased risk of respiratory infection in aged individuals (Schneider et al., 2021). Studies have shown that aging impairs the ability of macrophages and/or neutrophils to recognize pathogens and to produce proinflammatory cytokines/chemokines, due to the age-dependent loss of toll-like receptor (TLR) expression in these myeloid cells (Renshaw et al., 2002; Cho et al., 2018). To gain insight into the immune-deficient phenotype of pulmonary fibroblasts, we analyzed the age-related changes in a panel of innate immune receptors. Violin plots showed that pFBs expressed high level of *Il1r1* but not other TLRs, whereas macrophages and neutrophils expressed higher level of *Tlr2* (Figure 6A; Supplementary Figure S6A). Furthermore, *Il1r1* expression and a panel of IL1 pathway downstream chemokine gens (*Cxcl1, Cxcl12*) was notable lost in the 1.5 years aged pFBs compared to the 2 months young pFBs, accompanied with age-related loss of the expression of lipogenesis genes and gain of fibrosis genes (Figure 6B).

TGFβ plays a central role in the development of idiopathic pulmonary fibrosis by regulating several cellular processes, such as myofibroblast differentiation, extracellular matrix deposition, and cell senescence (Saito et al., 2018; Ye and Hu, 2021), and activation of TGF-β signaling has been linked with the development of several aging-associated disorders (Tominaga and Suzuki, 2019). Therefore, we next investigated whether TGFβ treatment in young pFBs may lead to the development of the aging phenotypes observed by sc-RNAseq. As shown in Figures 6C–N; Supplementary Figure S6B, TGFβ treatment on young primary pulmonary fibroblasts (isolated from 2-month-old mice) significantly increased the expression of fibrosis and/or myofibroblasts (A*cta2, Col1a1, Postn, Eln, Tnc*), inhibited the expression of genes related to lipogenesis (*Pparg, Apoe, Ly6a*) as well as immune response (*Il1r1, Cxcl1, Cxcl5, Cxcl12, C1ra*). These *in vitro* changes are highly consistent with the age-dependent changes of pFBs shown by scRNAseq, indicating that age-dependent activation of the TGFβ pathway may play a role in driving pulmonary fibroblast aging. Future study is still needed to validate the role of TGFβ pathway in driving the development of the fibrotic, immune-deficient, and lipodystrophy phenotypes in pulmonary fibroblasts during aging.

## Conclusion

We present a single-cell transcriptomic atlas of lung tissues derived from mice at various stages of aging, uncovering substantial shifts in fibroblast differentiation status, fibrotic phenotype, and immune regulation potential throughout the aging process. These changes of pulmonary fibroblasts may lead to age-dependent increase of risk for pulmonary fibrosis. Our *in vitro* data suggest that TGFβ may play a role in promoting the development of the fibrotic, immunodeficient and lipodystrophic phenotypes during the aging process of pulmonary fibroblasts. The findings of our study are poised to advance the development of biomarkers, diagnostic techniques, and targeted therapies for lung diseases associated with aging.

Together, our single-cell transcriptomic analysis provides an in-depth examination of an age-associated transcriptomic atlas for the lung fibroblast lineage, and establishes an intricate cell interaction network between pFBs and immune cells in mice across various stages of aging. These results may provide the foundation for in-depth research into aging-associated pulmonary pathologies.

## Discussion

In this study, we utilized single-cell transcriptomic sequencing to elucidate the diversity of cell types within lung tissues derived from mice at various ages, creating an inventory of marker genes for each cellular subtype, the transcriptional profiles and differentiation trajectories of pulmonary fibroblasts, and cell-cell interaction networks. We found that aged pFBs gained the characteristics of myofibroblasts, key contributors to pulmonary fibrosis (Wynn and Ramalingam, 2012), and this change may increase the risk of aged lung to pulmonary fibrosis development. Pulmonary lipo-fibroblasts, which can store lipid droplets within lung tissues, play an important role in maintaining lung homeostasis by involving in the production of surfactants by type II alveolar epithelial cells and alveolar development and regeneration associated with retinoic acid (vitamin A) storage (Tahedl et al., 2014). In contrast to age-related gain of fibrotic gene expression, the expression levels of lipogenesis-related genes including *Pparg*, *Plin2*, *Fabp1*, *Fabp4*, *Fabp5*, *Lpl*, and *Lipa* (Chen et al., 1998; Chen et al., 2012) as well as chemokines (Ccl2 and Cxcl12) were notably lost in aged pFBs compared to young pFBs as shown by our scRNA-seq data. These results suggest that pFBs may play critical roles in immune regulation, particularly warranting further investigation into their interactions with alveolar macrophages.

During the pathogenesis of pulmonary fibrosis, adipogenic fibroblasts have been reported as a significant source of activated myofibroblasts (Varisco et al., 2012). Our pseudotime analysis of cell lineage differentiation revealed the initial and final differentiation states of pFB through life-span, and indicates that lipofibroblasts may transdifferentiate toward myofibroblasts during aging. This age-related adipogenic to fibrotic switch of pFBs may increase the risk of fibrosis development upon lung injury and/or infections in aged lung tissues.

TGFβ signaling plays a central role in promoting the transition of lipofibroblasts to myofibroblasts during the pathogenesis of lung fibrosis (El Agha et al., 2017; Saito et al., 2018; Ye and Hu, 2021), and activation of TGFβ signaling has also been linked with the development of several aging-associated disorders (Tominaga and Suzuki, 2019). We have previously shown that age-dependent activation of TGFβ pathway in dermal fibroblasts leads to impaired adipogenic and antimicrobial innate immune response of dermal fibroblasts to invading bacteria, leading to increased susceptibility of aged mice to skin bacterial infection (Zhang et al., 2019). Here we found that TGFβ treatment in young pFBs not only induced the expression of fibrotic genes, but also inhibited IL1 and innate immune related genes as well as lipogenesis genes (Figures 6C–N). Future study is still needed to validate the *in vivo* role of TGFβ pathway in driving the development of aging phenotypes in pulmonary fibroblasts.

## Data Availability

The datasets presented in this study can be found in online repositories. The names of the repository/repositories and accession number(s) can be found below: https://www.ncbi.nlm.nih.gov/geo/query/acc.cgi?acc=GSE237757.

## Abbreviations

pFBs, pulmonary fibroblasts; IPF, idiopathic pulmonary fibrosis; scRNA-seq, Single-cell RNA sequencing; HE, hematoxylin and eosin; NB, new born; 2 M, 2 months years old; 10 M, 10 months years old; 18 M, 18 months years old; Postn, Periostin; BLM, bleomycin; FB, fibroblasts; Tdg, delta gamma T cells; Treg, regulatory T cells; NKT, natural killer T cells; Neu, neutrophil; Inter_MAC, interstitial macrophage; ALV_MAC, alveolar macrophages; ILC, innate lymphoid cells; Pneu, pneumocyte; EC, endothelial cell.
