# Supplementary material for: Deciphering the age-dependent changes of pulmonary fibroblasts in mice by single-cell transcriptomics
Source: Front Cell Dev Biol. 2023 Nov 29;11:1287133. doi: 10.3389/fcell.2023.1287133 (PMC10716426; doi:10.3389/fcell.2023.1287133)
Supplement: Supplementary file 2 [file DataSheet1.ZIP › 110623-supplemental figure and legend.pdf]

Fig1.Supplementary material

A.

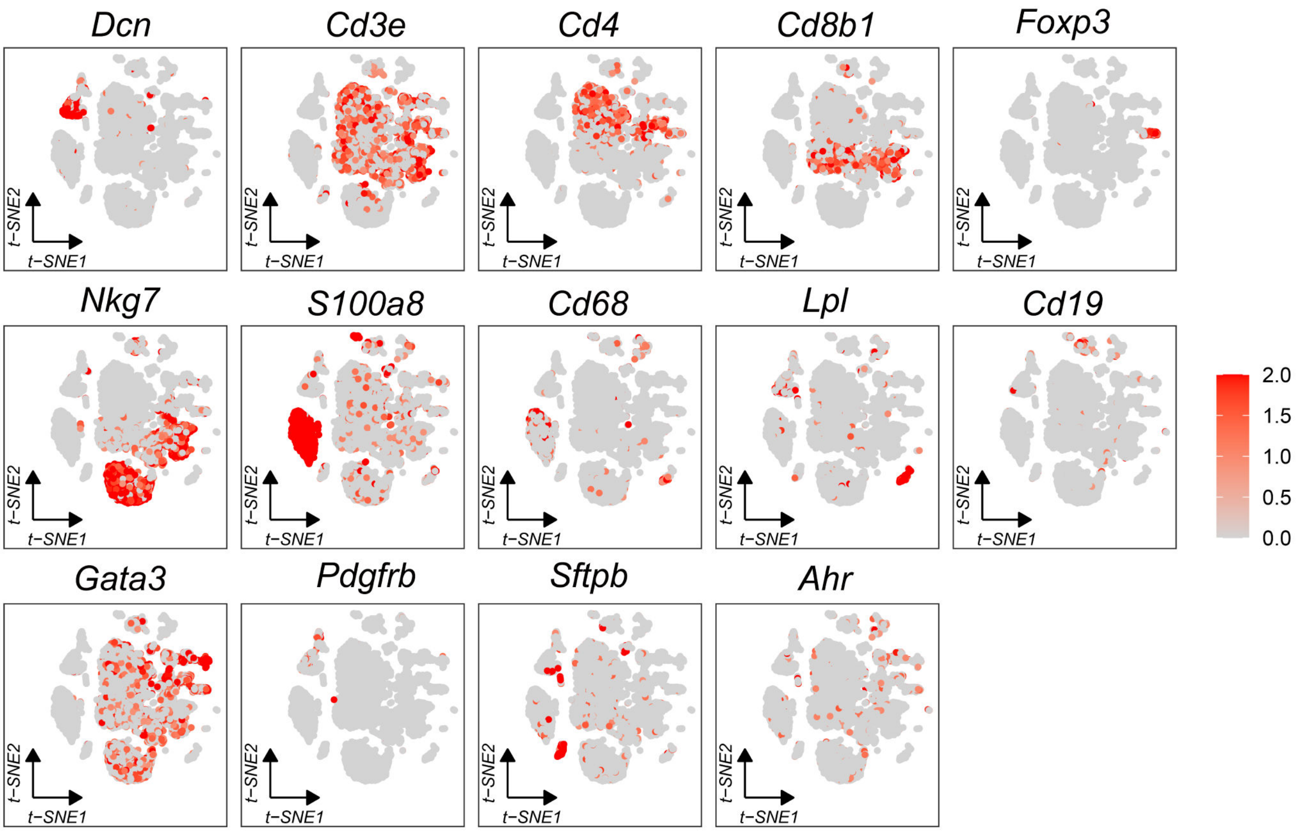

B.

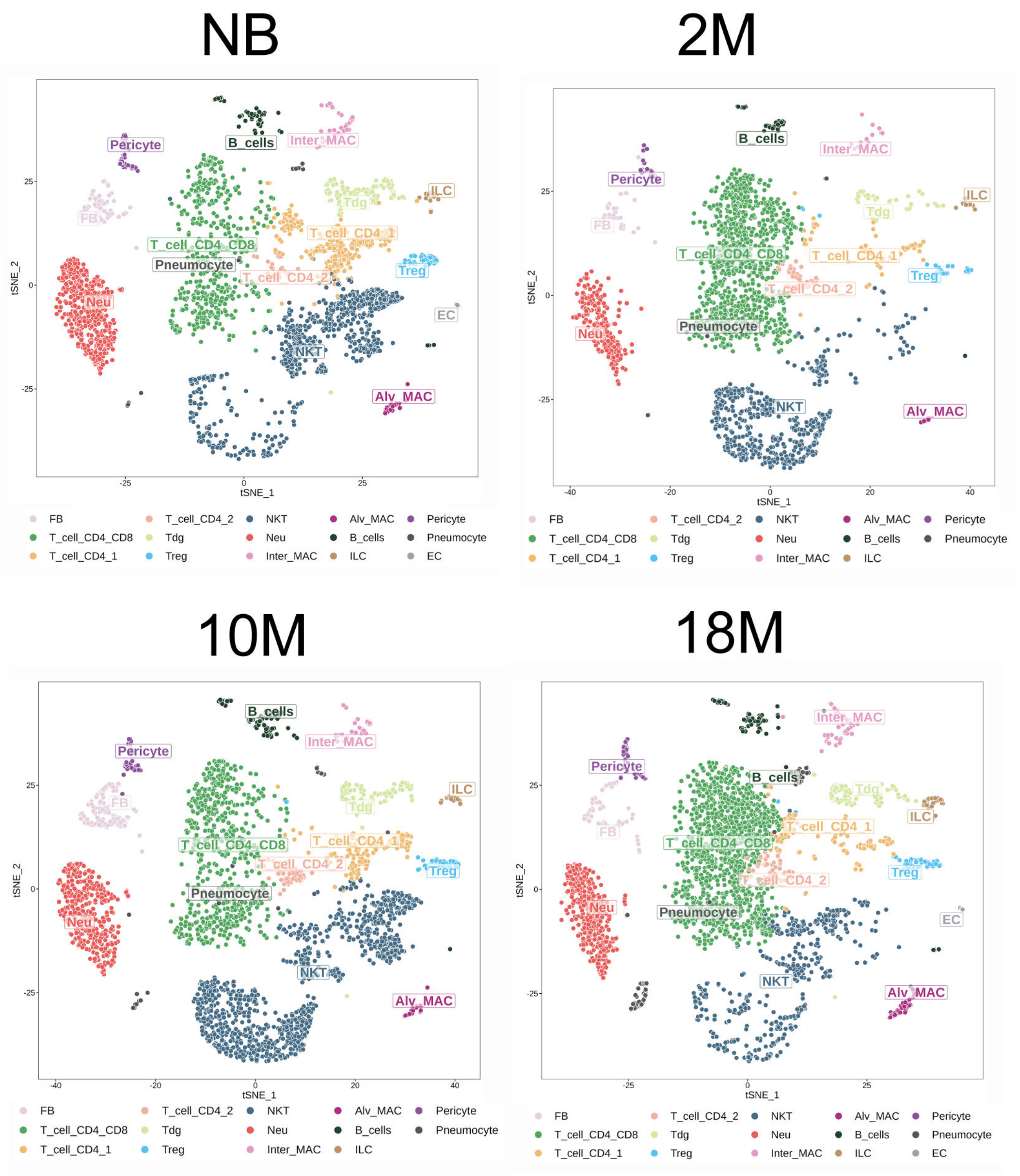

C.

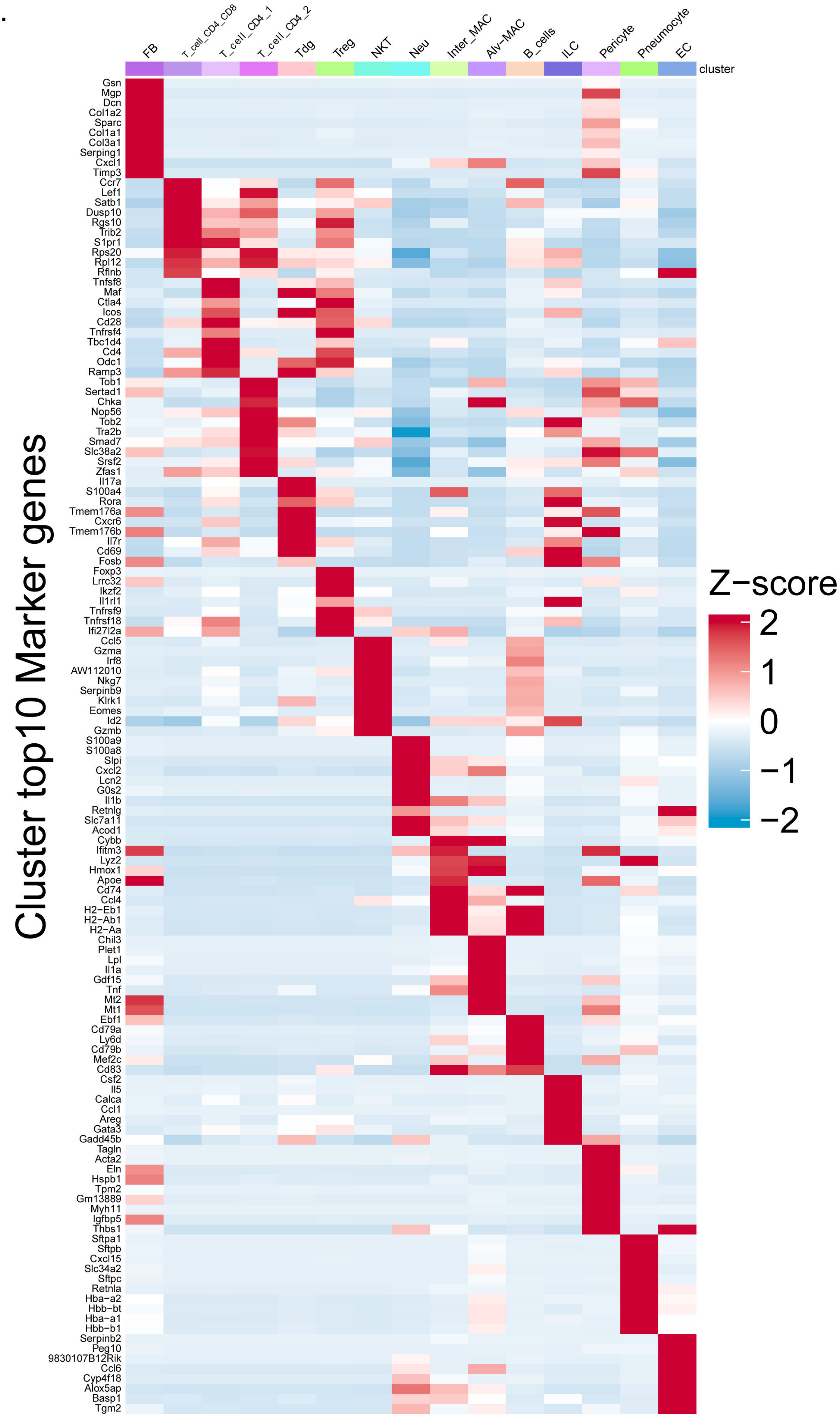

FB
T_cell_CD4_CD8
T_cell_CD4_1
T_cell_CD4_2
Tdg
Treg
NKT
Neu
Inter_MAC
Alv-MAC
B_cells
ILC
Pericyte
Pneumocyte
EC
cluster
Cluster top10 Marker genes
Z-score
2
1
0
−1
−2
Gsn
Mgp
Dcn
Col1a2
Sparc
Col1a1
Col3a1
Serping1
Cxcl1
Timp3
Ccr7
Lef1
Satb1
Dusp10
Rgs10
Trib2
S1pr1
Rps20
Rpl12
Rflnb
Tnfsf8
Maf
Ctla4
Icos
Cd28
Tnfrsf4
Tbc1d4
Cd4
Odc1
Ramp3
Tob1
Sertad1
Chka
Nop56
Tob2
Tra2b
Smad7
Slc38a2
Srsf2
Zfas1
Il17a
S100a4
Rora
Tmem176a
Cxcr6
Tmem176b
Il7r
Cd69
Fosb
Foxp3
Lrrc32
Ikzf2
Il1rl1
Tnfrsf9
Tnfrsf18
Ifi27l2a
Ccl5
Gzma
Irf8
AW112010
Nkg7
Serpinb9
Klrk1
Eomes
Id2
Gzmb
S100a9
S100a8
Slpi
Cxcl2
Lcn2
G0s2
Il1b
Retnlg
Slc7a11
Acod1
Cybb
Ifitm3
Lyz2
Hmox1
Apoe
Cd74
Ccl4
H2-Eb1
H2-Ab1
H2-Aa
Chil3
Plet1
Lpl
Il1a
Gdf15
Tnf
Mt2
Mt1
Ebf1
Cd79a
Ly6d
Cd79b
Mef2c
Cd83
Csf2
Il5
Calca
Ccl1
Areg
Gata3
Gadd45b
Tagln
Acta2
Eln
Hspb1
Tpm2
Gm13889
Myh11
Igfbp5
Thbs1
Sftpa1
Sftpb
Cxcl15
Slc34a2
Sftpc
Retnla
Hba-a2
Hbb-bt
Hba-a1
Hbb-b1
Serpinb2
Peg10
9830107B12Rik
Ccl6
Cyp4f18
Alox5ap
Basp1
Tgm2

FIGURE S1. Commencing with the classification of cells within aging mouse lung tissue via scRNA-seq. **A.** Feature plots showing the expression of indicated marker genes. **B.** tSNE projection of sequenced mouse cells, showing the partitioning of 15 cell types by aging sample. **C.** Heatmap showing the top10 marker genes in aging mouse lung each subpopulation by z-score normalization analysis. Color scale of red to blue indicates z-score.

Fig2. Supplementary material

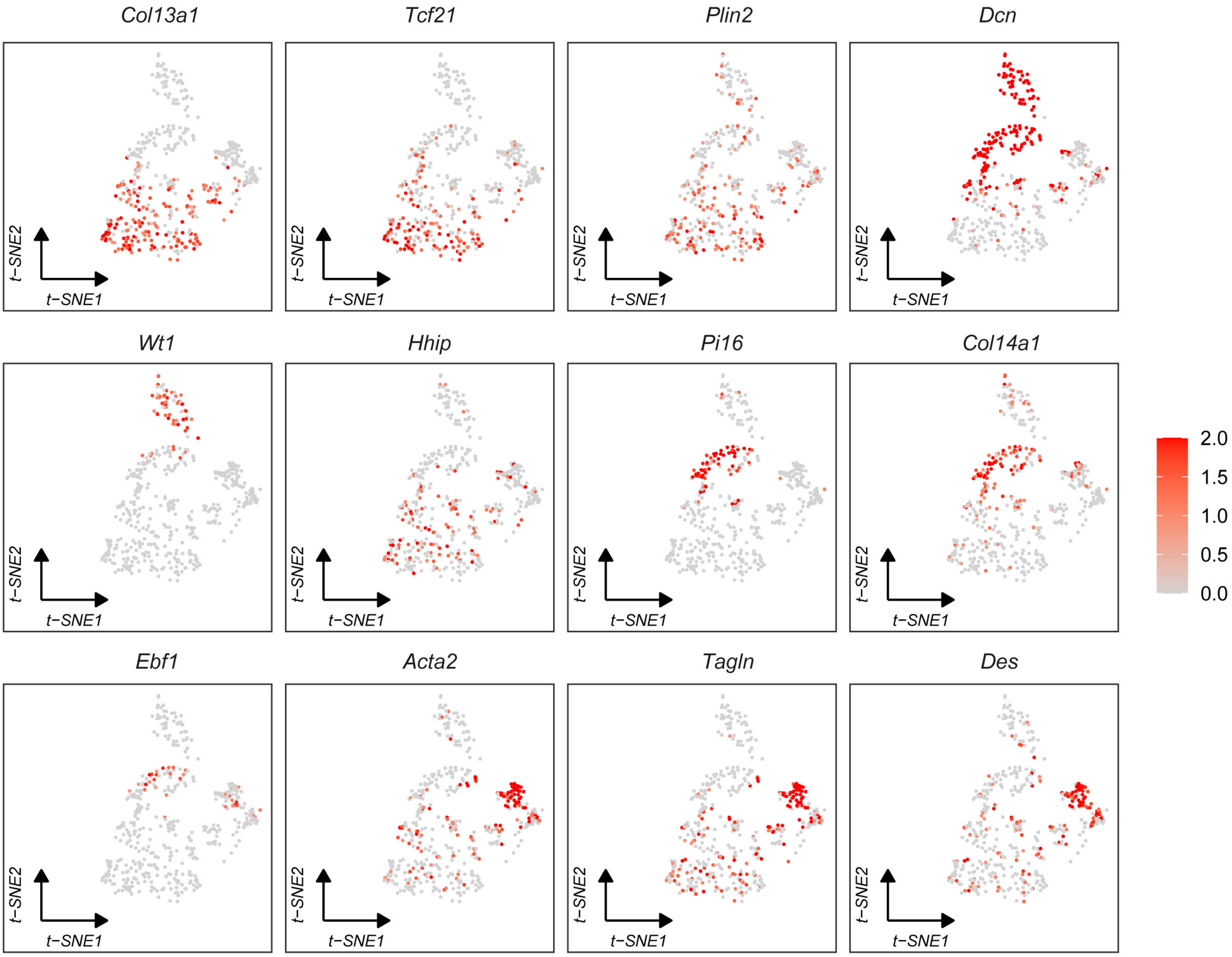

FIGURE S2. Representative marker genes for *Pdgfra*$^+$ pFB subclusters. Feature plots showing the expression of subcluster marker genes for the *Pdgfra*$^+$ pFBs from the BLM-treated mouse lung sc-RNAseq database (GSE129605).

Fig3. Supplementary material

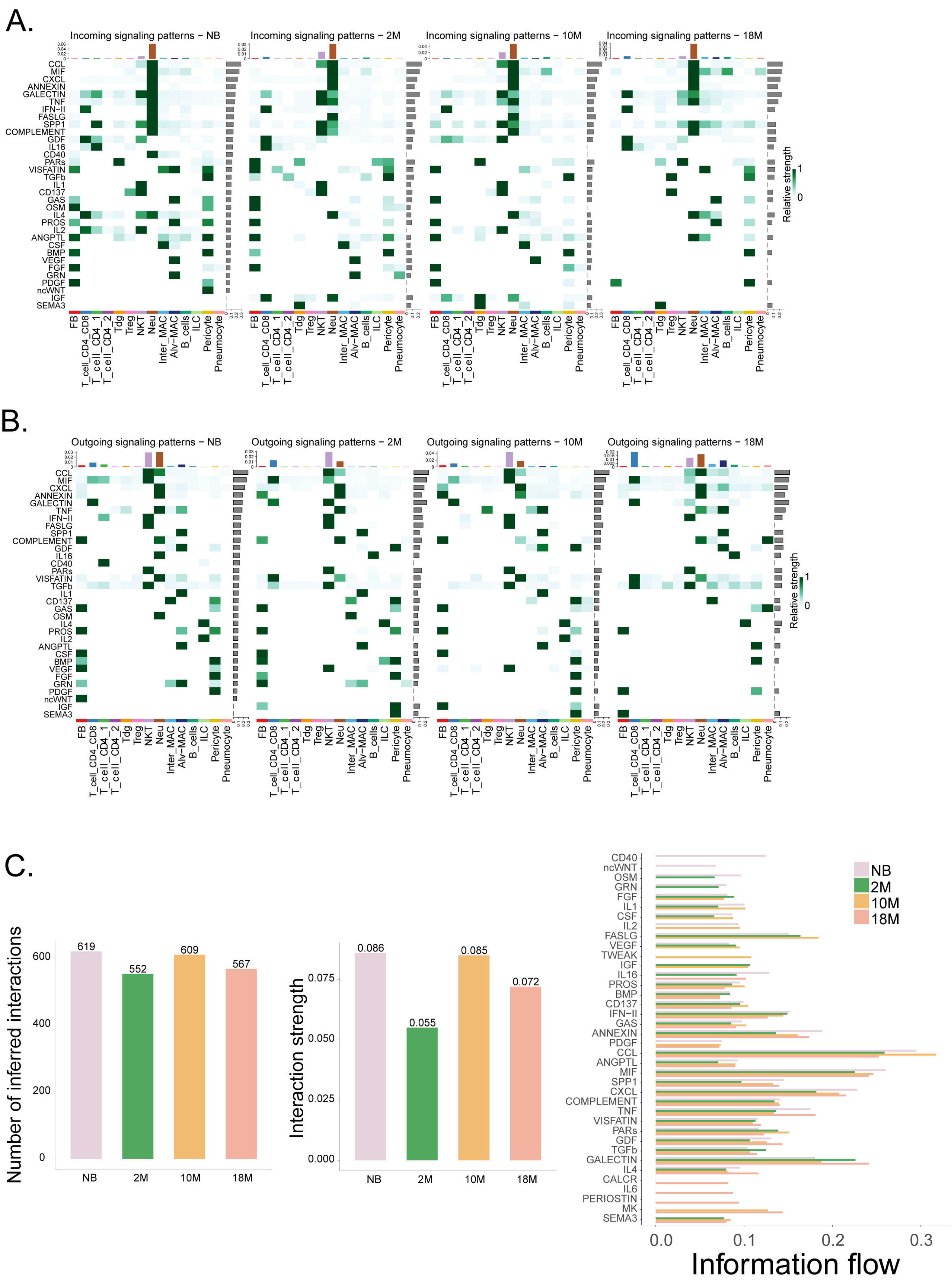

FIGURE S3 Anticipating the communication dynamics between fibroblasts and immune cells at various stages of aging. **A.** Heatmap for the differential network analysis only works for pairwise incoming datasets. **B.** Heatmap for the differential network analysis only works for pairwise outgoing datasets. **C.** Bar chart for comparison the number of interactions and interaction strength among different cell populations. **D.** Bar chart for comparison of the significant ligand-receptor pairs between 2M and 18M.

Fig4. Supplementary material

A. CCL total singaling pathway-2M   CCL total singaling pathway-18M

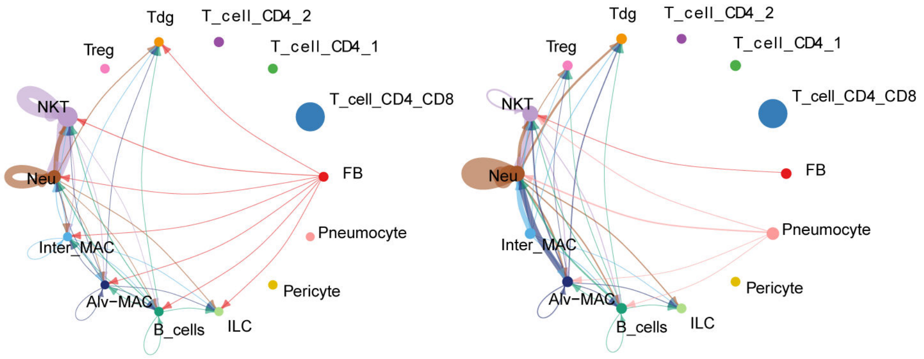

B. CXCL total singaling pathway-2M   CXCL total singaling pathway-18M

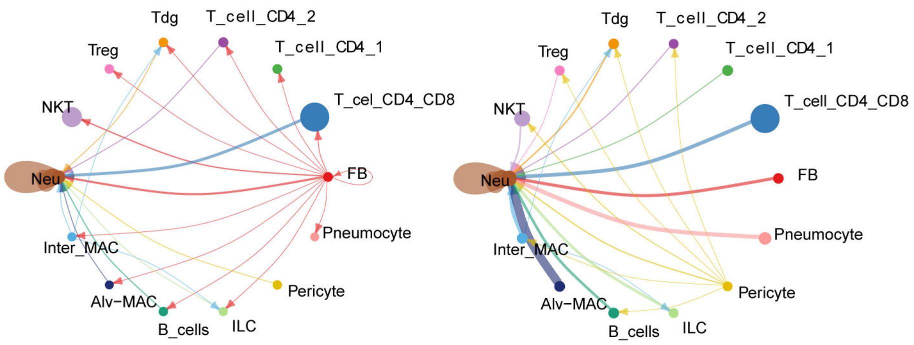

FIGURE S4 Anticipating the communication dynamics between fibroblasts and immune cells at various stages of aging. **A.** Circle and hierarchy plot showing total, 2M, 18M cell-cell communication in PROS signaling pathway (left, middle, right). **B.** Circle and hierarchy plot showing total, 2M, 18M cellcell communication in IGF signaling pathway (left, middle, right). **C.** Circle and hierarchy plot showing total, 2M, 18M cell-cell communication in Complement signaling pathway (left, middle, right).

Fig5. Supplementary material

A.

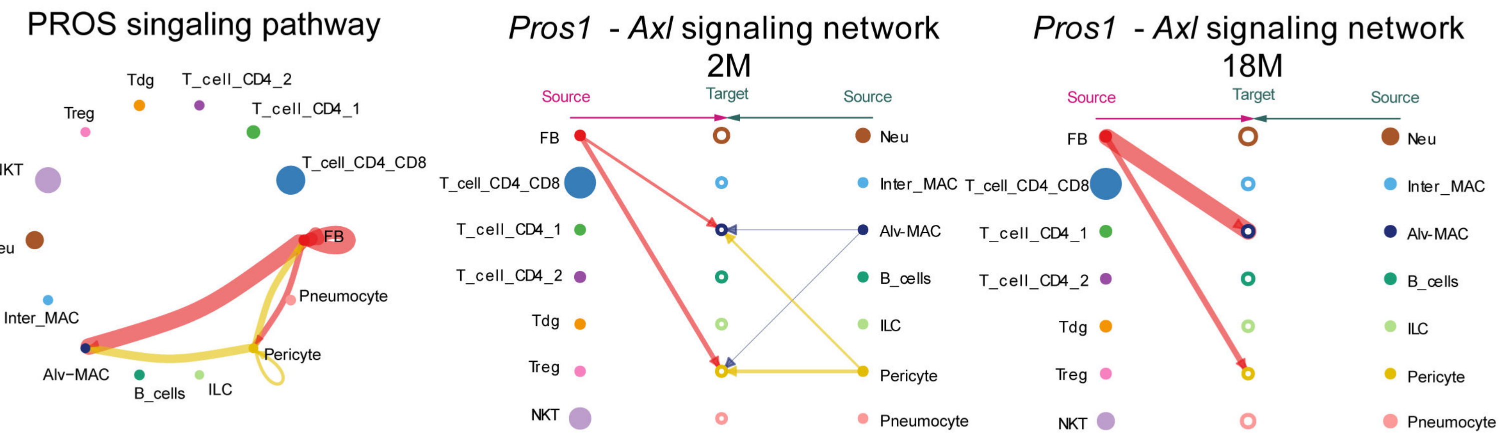

B.

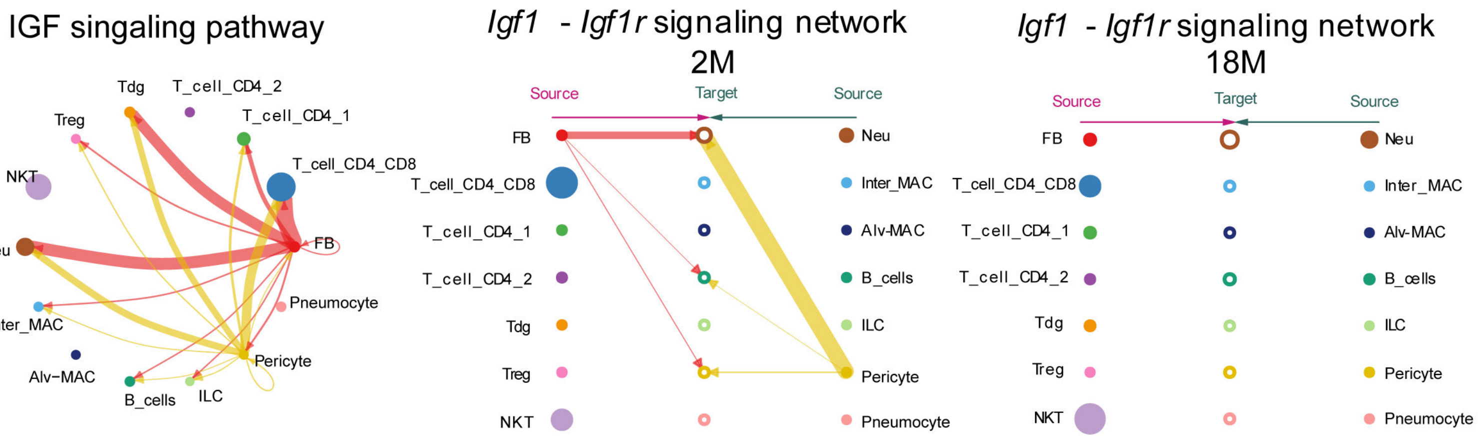

FIGURE S5 Anticipating the communication dynamics between fibroblasts and immune cells at various stages of aging. **A.** Circle and hierarchy plot showing 2M, 18M cell-cell communication in CCL signaling pathway (left, middle, right). **B.** Circle and hierarchy plot showing 2M, 18M cell-cell communication in CXCL signaling pathway (left, middle, right).

Fig6. Supplementary material

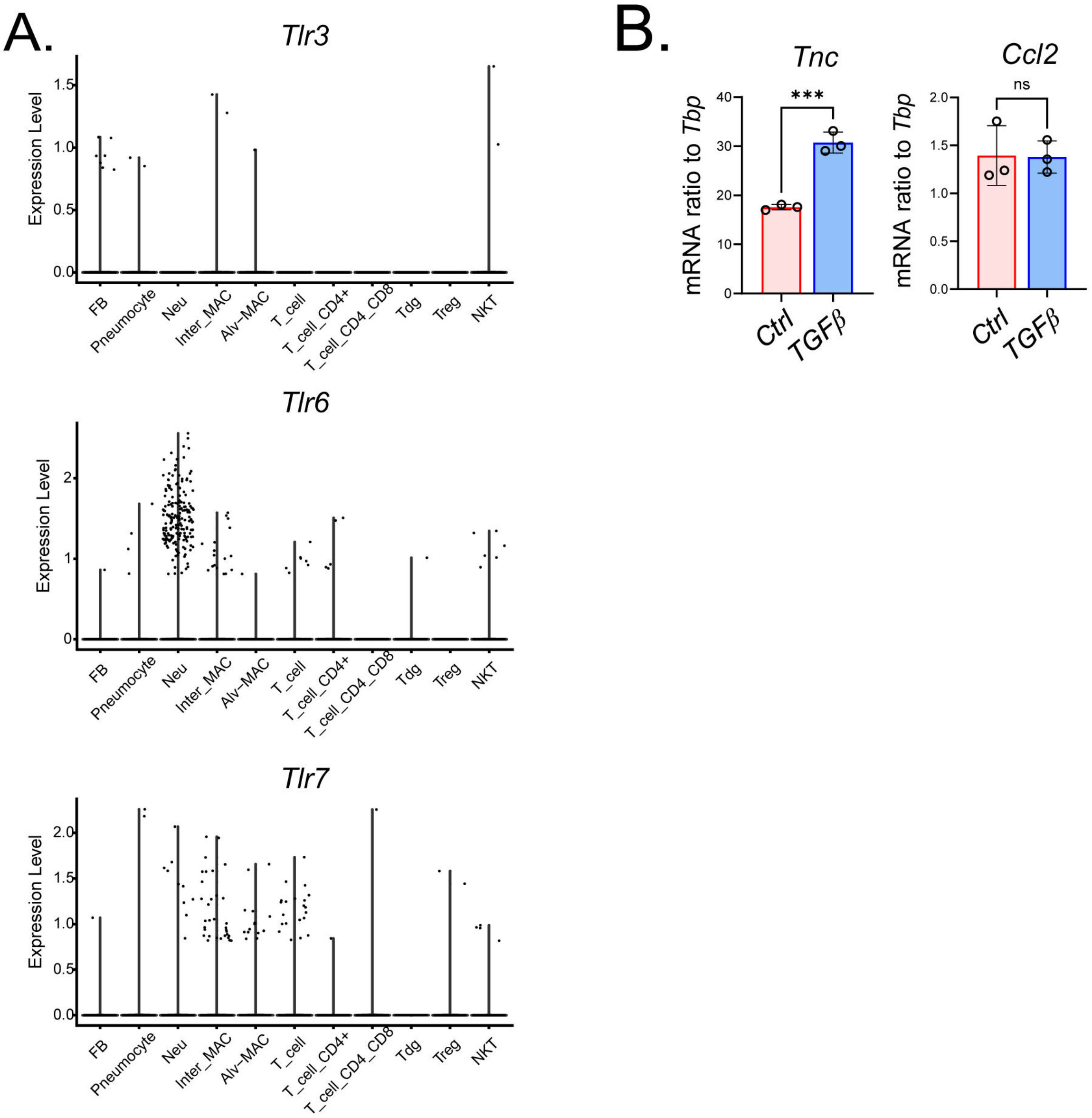

FIGURE S6 Activation of TGFβ in cultured pulmonary fibroblasts induces the transcriptional signature of aging. (A). Violin plots showing the expression of indicated TLRs in various pulmonary cell clusters as shown. **(B).** Primary pulmonary FBs were treated with recombinant mouse TGFβ2 at a concentration of 3 ng/ml for 2 days and then subjected to qRT-PCR of *Tnc* and *Ccl2* (n = 3/group). All error bars indicate mean ± SEM; *p < 0.05, **p < 0.01, ***p < 0.001.
